# Time is money: general practitioners’ reflections on the fee-for-service system

**DOI:** 10.1186/s12913-024-10968-3

**Published:** 2024-04-15

**Authors:** Kristian B. Kraft, Eivor H. Hoff, Magne Nylenna, Cathrine F. Moe, Arnstein Mykletun, Kristian Østby

**Affiliations:** 1 Cluster for Health Services Research, Norwegian Institute of Public Health, Oslo, Norway; 2 Institute of Health and Society, University of Oslo, Oslo, Norway; 3 Department of Community Medicine, UiT – The Arctic University of Norway, Tromsø, Norway; 4 Office of the Auditor General of Norway, Oslo, Norway; 5 Centre for Work and Mental Health, Nordland Hospital Trust, Bodø, Norway; 6 Faculty of Nursing and Health Sciences, Nord University, Bodø, Norway; 7 Centre for Research and Education in Forensic Psychiatry and Psychology, Haukeland University Hospital, Bergen, Norway; 8 Løkkegården GP Medical Centre, Ski, Norway

**Keywords:** General practice, Remuneration, Fee-for-service, Gatekeeping, Incentives, Consultation length

## Abstract

**Background:**

Fee-for-service is a common payment model for remunerating general practitioners (GPs) in OECD countries. In Norway, GPs earn two-thirds of their income through fee-for-service, which is determined by the number of consultations and procedures they register as fees. In general, fee-for-service incentivises many and short consultations and is associated with high service provision. GPs act as gatekeepers for various treatments and interventions, such as addictive drugs, antibiotics, referrals, and sickness certification. This study aims to explore GPs’ reflections on and perceptions of the fee-for-service system, with a specific focus on its potential impact on gatekeeping decisions.

**Methods:**

We conducted six focus group interviews with 33 GPs in 2022 in Norway. We analysed the data using thematic analysis.

**Results:**

We identified three main themes related to GPs’ reflections and perceptions of the fee-for-service system. First, the participants were *aware of the profitability* of different fees and described potential strategies to increase their income, such as having shorter consultations or performing routine procedures on all patients. Second, the participants acknowledged that the fees might *influence GP behaviour*. Two perspectives on the fees were present in the discussions: fees as incentives and fees as compensation. The participants reported that financial incentives were not directly decisive in gatekeeping decisions, but that rejecting requests required substantially more time compared to granting them. Consequently, time constraints may contribute to GPs' decisions to grant patient requests even when the requests are deemed unreasonable. Last, the participants reported challenges with *remembering and interpreting* fees, especially complex fees.

**Conclusions:**

GPs are aware of the profitability within the fee-for-service system, believe that fee-for-service may influence their decision-making, and face challenges with remembering and interpreting certain fees. Furthermore, the fee-for-service system can potentially affect GPs’ gatekeeping decisions by incentivising shorter consultations, which may result in increased consultations with inadequate time to reject unnecessary treatments.

**Supplementary Information:**

The online version contains supplementary material available at 10.1186/s12913-024-10968-3.

## Background

There are different ways to remunerate general practitioners (GPs) within publicly funded health services. The three most common types of remuneration include fee-for-service, capitation, and fixed salary. The different types of remuneration stimulate different kinds of GP behaviour [1, 2]. Fee-for-service incentivises GPs to increase the volume of health services, e.g., by reducing consultation length and increasing the number of visits, or by performing more procedures. It may also be used to influence the volume of specific procedures by increasing or decreasing the incentive. On the other hand, capitation incentivises GPs to enrol more patients on their list and keep them satisfied. Fixed salary does not provide any direct financial incentives for specific GP behaviours, often leading to lower volume of health services provided.

In Norway, primary health care is the responsibility of municipalities, and all residents have the right to be listed to a specific GP of free choice. GPs are mainly publicly funded and they have a gatekeeping role in referrals to specialist services, sickness certification, and prescriptions. In general, GPs in the GP scheme are remunerated in one of two ways: a combination of fee-for-service and capitation, or primarily through fixed salary. The majority of GPs (82%) are self-employed and receive remuneration from fee-for-service (70%) and capitation (30%) [3, 4]. A minority of GPs (18%) are employed by municipalities, with approximately half receiving a fixed salary and the other half receiving a fixed salary with additional forms of remuneration [3].

The fee-for-service system in Norway is operationalised through the *fee system* [5], which consists of 220 specific procedures (services) that have corresponding remuneration (fee). Fixed salary GPs use the fee system without receiving the remuneration. Instead, municipalities receive this generated fee income. This system includes fees for consultations, phone contacts, extra time used and various procedures and activities. It is a trust-based system and the government agency responsible for reimbursing GPs (HELFO) assume that all reimbursement claims are correct [6]. However, they are also conducting automatic controls of claims, and follow-up controls in instances where they suspect fraud. The number, content and amount of the fees are negotiated by the Ministry of Health and Care Service, the Norwegian Medical Association and The Norwegian Association of Local and Regional Authorities.

The problem of overdiagnosis and overtreatment has received increased attention over the years [7]. The international campaign *Choosing wisely* have drawn attention to the problem of unnecessary tests, treatments and procedures – where GPs in many cases serve as gatekeepers [8]. GPs' gatekeeping role are important for optimising prioritisation of resources and reduce treatments and interventions that may be harmful to patients. There is a rich literature on GPs’ gatekeeping role in Norway and GPs describe it as very challenging and uncomfortable [9–11]. Qualitative studies have found that GPs' gatekeeping decisions in sickness absence certification are, in practice, largely patient-driven and that GPs' role is limited to negotiations with the patients [9, 12, 13]. Studies have emphasised financial incentives as an aspect that may have impact on how the gatekeeping role is practiced [10, 12, 14].

Several studies show that the type of remuneration is associated with GPs’ gatekeeping decisions. Compared to salary and capitation, fee-for-service is associated with higher antibiotic prescription rates [15], higher rates of sickness certification [16, 17], fewer referrals to secondary care [2, 17, 18], and diverging results regarding prescriptions in general [1, 2]. A qualitative study from Canada reports that GPs believe that fee-for-service may lead to unnecessary overtreatment of patients and shorter consultations, which in turn may lead to more test, prescriptions, and referrals [19]. A study from Sweden finds that GPs perceive fee-for-service to stimulate shorter visits, up-coding, and skimming of healthier patients [20]. In Norway, GPs remunerated by fee-for-service and capitation have an estimated average consultation duration that is approximately three minutes shorter than fixed salary GPs (18 vs. 21 min) [21]. Other studies report that visit duration or time pressure may be related to antibiotic prescriptions [22–24], sick-listing [9], and addictive drugs [25].

To our knowledge, no studies have explored how GPs perceive a fee-for-service system where financial incentives affect their personal income. Furthermore, there is little knowledge on how GPs perceive their gatekeeping role in relation to the fee-for-service system. The aim of this study is to explore GPs reflections on and perception of the fee-for-service system, with a focus on how it might influence their gatekeeping decisions.

## Methods

We have conducted six focus group interviews with 33 GPs across Norway between September and November 2022. We contacted approximately 50 GP offices by phone and sent an invitation email. These offices had at least 4 GPs and were located in all five major geographical regions of Norway (Northern, Central, Western, Southern, and Eastern Norway) with large urban/rural variation. We received response from six offices, and the participating GPs were remunerated with NOK 2000 for participation for two hours. The six focus group GP offices were in Northern, Eastern or Western Norway. Two GP offices were in a municipality with more than 100,000 inhabitants, two were located in a municipality with 10,000–100,000 inhabitants, and two were located in municipalities with fewer than 10,000 inhabitants. The median age of the participants was 42 years, 61% were women, and 76% were certified specialists in general practice. Four of the offices had self-employed GPs who were remunerated by a combination of fee-for-service (70%) and capitation (30%). At one of the offices, the GPs were employed by the municipality and had a fixed salary, while one office had GPs with both types of employment and remuneration models. Table 1 show this information for each GP office. There is no relation between participant numbers used in the results and information in Table 1.

**Table 1:** Descriptive information on participating GPs by GP office

GP office No	N	Mean age	Female	Specialist	Inhabitants in municipality	Employment
1	5	40	60%	60%	< 10 000	Self-employed and employed by the municipality
2	6	39	67%	50%	< 10 000	Employed by the municipality
3	5	45	60%	80%	10 000 – 100 000	Self-employed
4	5	41	80%	80%	10 000 – 100 000	Self-employed
5	8	44	63%	88%	> 100 000	Self-employed
6	4	40	40%	80%	> 100 000	Self-employed

We chose to conduct focus groups because it is a suitable method for gathering variation in perspectives on a topic [26] and detecting group norms and values [27]. Also, it enables us to observe the interaction between participants that emerge during the interview [28]. We have developed a semi-structured interview guide based on literature, previous knowledge, and topics relevant for a larger project in which this study is a part of. It followed a similar structure in all interviews, although it underwent a minor revision after the initial interview and switched some questions in the interview with fixed salary GPs. The questions we asked concerned structuring of the working day, doubts concerning correct use of fees, work that is perceived as adequately/inadequate compensated, and time usage of gatekeeping decisions. A full interview guide is found in the supplementary file 1. Some of the questions were asked as yes/no questions to first establish whether the GPs agreed/disagreed with a statement, followed by open-ended questions asking the participants why or ask them to give examples to gain insight into their perceptions and opinions. Data collection for this study was conducted concurrently with another study on GPs’ gatekeeping role in sickness absence certification, allowing for efficient gathering of data for both studies [12]. In order to assist non-Norwegian speaking readers with information on which specific fee codes and their corresponding wording that are relevant at the time this study was conducted, we have included a list of fees mentioned in supplementary file 2.

The interviews were conducted by the first and second authors, except for one interview where only the second author was present. The interviews were audio recorded and automatically transcribed by Whisper [29]. This program transcribed the interviews with some mistakes, and the first author corrected the transcriptions. The first author conducted the analysis and coded the interviews using Braun & Clarke’s thematic analysis [30]. This method is divided into six phases: The first phase is about familiarizing oneself with the data, which was done during the process of correcting the transcriptions and re-reading the material. Second, we identified codes of interest across the interviews. Third, several themes and sub-themes representing a patterned response or important meaning within the data were identified. In the fourth and fifth phases, these themes were iteratively reviewed in terms of scope, content, and names. In these phases, the co-authors were involved in discussions. This iterative process of reviewing themes was conducted to produce coherent findings to the research questions while also reflecting variation among the participants. The last and sixth phase of the analysis was done in the writing process where example quotes had to be selected, and the overall structure had to be decided.

In the analysis, we were mainly interested in the self-employed GPs since they receive remuneration and therefore have financial incentives from the fee system. Because fixed salary GPs use the fee system without receiving remuneration, we wanted to include them in the data material as well. However, since we only have one interview with a full group of fixed salary GPs, we are not able to compare them directly in the analysis. For some subthemes, we have therefore included a perspective from the fixed salary GPs without explicitly comparing their statements to statements by self-employed GPs. This study is part of a project financed by the Research Council of Norway (#303583) with approval from the Regional Committees for Medical and Health Research Ethics (#210548). The Norwegian Institute of Public Health has conducted a data protection impact assessment of the data used. All participants received information about the study and have given consent to participate.

## Results

We identified three themes related to GPs’ reflections on and perception of the fee-for-service system, with a focus on GPs’ gatekeeping decisions: awareness of profitability, influence behaviour, and remembering and interpreting fees.

### Awareness of profitability

The first theme we have identified is GPs awareness of profitability. In general, the participants appeared to be aware of the profitability of the fee system. Participants expressed this awareness in various ways, either by recognizing the profitability of specific procedures, discussing different types of adaptation strategies that could increase their income, or expressing dissatisfaction with work that is not reimbursed by the fees. Although the participants showed an awareness of profitability, they did not express a large interest or need in maximising their fee income. This is exemplified by the following quote:

It's very rare that I think so thoroughly about what this will lead to. For the most part, we don’t think much about it, except right after the consultation. So, we don’t really dwell on the fee system. If you work normally and sensibly, you generally have a decent financial situation. So, you don’t need to make a huge effort in one direction or the other to avoid going bankrupt, at least. (#14, male GP specialist, self-employed)

The degree of awareness varied among the participants. Some seemed to have little interest or understanding of the profitability of the system, and they expressed themselves in a same vein as the GP in the quote above. Others showed more awareness of profitability, for instance, they could provide several examples of profitable fees and strategies.

#### Profitable fees

Several participants highlighted specific fees as particularly profitable. For example, the fee for removing warts was mentioned as highly profitable. This fee was considered profitable because it is possible to repeat it up to 8 times for each wart, and earn, according to the GPs, disproportionately much considering the work.

Cryosurgery, or wart treatment, is where you earn disproportionately well for something that is so simple and requires so little mental energy. (#16 female GP specialist, self-employed)

Additionally, some participants described electronic consultations conducted in the evening as another profitable fee. This can be used for consultations with video, but also for written communication with patients online. A participant expressed ambivalence for charging simple electronic messages:

I can charge for what I do, but it may seem a bit much to charge 340 kroner [29 Euro]. (#28, male GP specialist, self-employed)

#### Unprofitable fees and unpaid work

Conversely, the participants described tasks where the fees were low or where the tasks were not directly compensated in the fee system. Many expressed frustration at these tasks, considering them as unpaid work. Administrative tasks conducted in the evening were particularly emphasised as frustrating:

There are so many of those evening tasks I do that I don’t get paid for. It’s just expected to be included in some of the consultations. (#25, female GP specialist, self-employed)

#### Profitable strategies

The participants described potential strategies to increase income. These strategies included reducing the standard duration of consultations to increase volume, hiring nurses to perform routine procedures on all patients, advertising for specific procedures of profitable fees, or increasing list size and conduct numerous electronic consultations in the evening and morning. A participant noted:

There are probably some who get quite creative when using the fees, and what can I say, whether they are good at it, I don’t really know, but they can certainly earn a lot by stretching the limits. (#30, male GP specialist, self-employed)

The participants perceived several of these strategies as problematic and did not report deliberately using these strategies to increase their income. When such strategies were mentioned, they either described it as a theoretical possibility within the system or as something they have heard or observed other GPs have done. A participant described her experiences with former colleagues at an out-of-hours clinic:

I used to work at X out-of-hours clinic, and there were some doctors there who really pursued those quick consultations, like 10 per hour or whatever it was. They used a lot of fees, and it’s easy to use the fees if you have many straightforward cases, nurses handling everything, doing everything for you, and you don’t really care about being thorough. They overused the fees. (#12, female GP, under specialisation)

This kind of overuse of fees that seemed economically motivated was frowned upon by the participants and was perceived as rare exceptions. The focus group consisting of participants remunerated by fixed salary did believe they were less aware of the profitability, as their personal income was not affected by their use of fees. In response to a question about the advantages of fixed salary, a GP responded:

That you don’t work based on the fees, like some might do, that you are driven by earnings. (#3, male GP, employed by the municipality)

### Influence behaviour

The second theme we have identified is the fee-for-service system’s influence on GPs behaviour. In general, participants reported that their treatment decisions were primarily driven by medical indication and professional judgement rather than personal profitability.

We try to do things based on medical indication, not because there is a lot of money to gain. (#10, female GP specialist, self-employed)

#### Fees as compensation vs fees as incentives

We identified two types of perspectives that the participants have of the fees. The first perspective perceives fees as a *compensation*, i.e., that the GPs perform their work independent of the fees and are reimbursed for the work they have done. In this perspective, the fee-for-service system is seen as a pragmatic tool to receive income and is not believed to influence behaviour:

The fee system is probably not decisive, like, okay, I’ll do it this way because I’ll earn more. That rarely happens. It’s just something that needs to be done, and then we can charge for it. (#30, male GP specialist, self-employed)

The second perspective present in the discussions perceives fees as *incentives,* suggesting that the purpose of the fees is to influence behaviour. In this perspective, fees are seen as a management tool to encourage specific medical activities or prioritize certain political goals. The participants reported that the introduction or increase in fees for specific services, e.g. home visits or systematic medication reviews, led to greater usage of those services. The following quote exemplifies this perspective:

I think I would put it like this: what we do can generally be influenced by increasing or decreasing the fee. (#18, male GP specialist, self-employed)

Most participants held both perspectives, and saw them as complimentary understandings of the fees. However, in some discussions, the two perspectives appeared more conflicting. The dialogue below illustrates this:

[The interviewers asked whether they think the fees influence behaviour]

#28: Yes, I think so.

#26: Huh, you do?

#28: To some extent, yes. Do you think people would have been doing systematic medication reviews if there wasn’t a good fee for it?

#26: It’s actually required by law that we should do systematic medication reviews. It’s in the regulations. So we have to do it. And so there’s a fee for it.

(#28, male GP specialist, self-employed) (#26, male GP specialist, self-employed)

This discussion highlights the clash between different perspectives. Participant 28 believes that fees serve as an incentive for GP behaviour, while participant 26 opposes this idea. Participant 26 argues that GPs perform tasks considered good medical practice – and the fees serve as compensation for those tasks. While participant 26 challenges the idea of fees as incentives, most participants did not oppose either perspective.

#### Do incentives challenge the medical judgement?

Whether the participants emphasised fees as compensation or as incentives depended on the situation. When viewing fees as incentives, they tended to highlight positive or neutral effects based on their own experiences. For example, they mentioned their willingness to provide an extra effort to have more consultations:

If I’m thinking about whether to do it or not, and then I remember it’s paid for, it’s a bit like taking on an extra patient, you know. You wouldn’t bother if it’s not paid, but if it is paid, maybe you could do it. (#20, male GP specialist, self-employed)

While participants could discuss profitable strategies used by other GPs that had adverse effects, such as overtreatment, they rarely talked about fees as incentives when discussing adverse effects of their own behaviour. Most participants believed that their medical judgement counterbalanced the potential adverse effects of incentives in a large extent. A participants noted:

To make a medically inferior choice to earn more, no, that’s not an option. (#30, male GP specialist, self-employed)

However, one participant reflected on his own usage of the fee for 24 hour registration of blood pressure, which he considered profitable. The profitability of the fee caused doubt in his own medical judgement, leading him to speculate if he overtreated patients due to the incentives:

24-hour blood pressure registration, which is a simple procedure, is very well paid. This actually means that every time I do this procedure, I feel guilty because you start to doubt your medical judgment. Am I doing this because it’s well paid, or am I doing this because I genuinely think it’s the right thing to do? I often think it’s because I find it relevant, but then I see that I have more 24-hour registrations than many of my colleagues. That’s a bit exhausting with the fee system. (#15, male GP specialist, self-employed)

GPs remunerated by fixed salary believed this payment model enabled them to make better medical choices:

We feel that we provide better healthcare by being remunerated by fixed salary rather than chasing money, “quickly in, quickly out”. (#2, female GP specialist, employed by the municipality)

#### Gatekeeping decisions

For gatekeeping decisions, such as handling patient requests for referrals, antibiotics, addictive drugs, or sickness certification, participants tended to highlight fees as compensation. Most argued that the fees did not influence their decision to accept or reject these requests. They emphasised that other factors, such as medical judgement, were more important than economic incentives. Additionally, there was little or no direct economic incentive for referring or sick list patients.

However, participants reported that many patients either demanded or had high expectations for their treatment requests to be granted. Even when participants did not believe it was beneficial to accept a patient request for sickness certification or a referral, they found it difficult to reject them. They found rejections to be stressful and time-consuming, as it required significant more time and effort to explain why the requested treatments were unnecessary or not beneficial for the patients.

It is definitively most time-consuming if you have to reject someone. Because then you have to explain your choice, and then you have to argue, and you have to stick to it. The easiest thing is always just to say yes to what the patient is asking for. (#3, male GP, employed by the municipality)

As a result, many reported that the lack of time was a contributing factor in their decision to accept requests that they did not consider beneficial for patients’ health. When asked whether time shortage influences their decision, a participant responded:

Yes, it does. If you’re in a hurry or having a bad day, we give in. That’s how it is. After all, we’re only human. (#7, female GP specialist, self-employed)

Considering that having many short consultations was one of the most profitable strategies emphasised by the participants, it seems that the system incentives GPs to be more lenient gatekeepers. A GP described this logic:

Let’s say, if you had a day where you rejected everything, you would end up with significantly less money than if you took a lot of blood tests and made referrals. (#11, male GP specialist, self-employed)

### Remembering and interpreting fees

The third theme we identified concerned remembering and interpreting fees.

#### Remembering

In general, the participants reported that the fee system was comprehensible and did not significantly hinder or benefit their everyday practice. However, they pointed out two challenges when using the fee system. First, they reported that it was challenging to remember or know all the fees.

In general, the fee system is okay to adhere to. But most of us have had such an “aha” moment where we suddenly discover a fee we’ve never used before. (#11, male GP specialist, self-employed)

With over 200 different fees, GPs need to make an effort to remember the fees and stay continuously updated with changes. While fees used regularly are easy to remember, they find it harder to remember fees that are less frequently used.

I become more productive when I go through that fee booklet now and then. Then I realize, ‘Oh, I don’t charge for this.’ I should have. And then, I might remember better for next time and take note of it. (#29, female GP specialist, self-employed)

#### Interpreting

The second way the fee system challenged GPs was through their interpretation of how fees should be applied. Some fees have a clear-cut definition with no room for interpretation, while others leave room for the GP’s interpretation. The participants mentioned several fees that they were uncertain in how to use, and that the threshold for usage of certain fees could be ambiguous. The fee for talking therapy was highlighted as one that could be hard to interpret:

When is it a conversation? When is it a moral support conversation? These doesn’t trigger any fees. And when does it qualify as a therapeutic conversation with a cognitive focus or whatever it says? (#13, male GP specialist, self-employed)

Remembering and interpreting fees become more challenging when fees are complex. Some fees have several detailed requirements that many of the participants find hard to fulfil in practice, such as fees for systematic medication review or smoking cessation.

And there are fees where the requirements are completely unrealistic, which means I don't actually use them. A good example is 2LD - systematic medication review, where it states that there should be at least 4 medications, and it cannot be combined with time fee 2CD, and it needs to be in line with the Directorate of health’s guidelines. In the Directorate of health’s guidelines, there are many, many things that need to be done, so the only time I use that fee is when they have exactly 4 medications, and I don't spend much time on it, or I try to conduct the reviews when the patient is in the office. Then it takes an hour or an hour and a half, and I'm still not in line with the Directorate of health’s guidelines. (#15, male GP specialist, self-employed)

#### Variation in interpreting and remembering fees

A consequence of a system where GPs need to remember and interpret fees is that GPs could underuse or overuse fees. The participants believed that there are large differences in the degree of to which GPs use the fees:

Yes, I believe we do it quite differently all the time, even though it may appear very intuitive. But it’s open to interpretation nonetheless, even though it’s just a fee. (#7, female GP specialist, self-employed)

Some GPs highlighted that variation in the interpretation of fees were related to the degree of awareness of profitability:

I see that when I am in discussion groups with other GPs, there are some who exploit or interpret the fees in their favour in order to earn more. (#3, male GP, employed by the municipality)

## Discussion

**Fig. 1:**
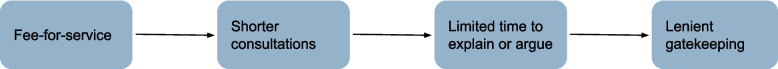
Illustration of proposed mechanism linking fee-for-service and lenient gatekeeping

This study found that GPs’ gatekeeping role can be challenged by the fee-for-service system, which incentivises shorter consultations and, in turn, provides less time to reject unnecessary treatments and interventions. The participants reported that many short consultations are profitable, and that shortage of time may contribute to granting patient requests, even those they consider unreasonable. This is because rejecting requests is more time-consuming than granting them. Therefore, it appears that the fee-for-service system may contribute to a more lenient gatekeeping practice through limited time to explain and argue with the patients in shorter consultations (Illustrated by Fig. 1).

This finding is supported by a study from Canada, but we provide a new mechanism [19]. The Canadian study emphasises that the mechanism linking shorter consultations and increased treatment is through incomplete case history, which in turn leads to more uncertainty and increased treatment. In our study, we find that shorter consultations limit GPs’ possibilities to explain why they consider the treatment unreasonable, which in turn leads to more lenient gatekeeping practices.

Our results indicate that the fee-for-service system requires both effort and conscientiousness from GPs to function as intended. The participants reported challenges with remembering and interpreting fees and expressed awareness of the profitability within the system. They perceive fees both as compensation for performed work and as an incentive that influences behaviour. When GPs do not remember fees, they do not receive compensation for performed work, and the fee-for-service’s effect on behaviour is likely to be limited. The fee-for-service system is largely a trust-based system, and therefore, how individual GPs interpret fees is partly based on their conscientiousness. Although none of the GPs we interviewed considered their own fee behaviour unethical, they could mention other GPs’ fee behaviour that they considered as a grey-zone interpretation or what they considered as unethical interpretation. Since GPs receive a solid income based on the fee-for-service system, they can be described as stakeholders in the system. Therefore, questions regarding the relationship between personal income and medical decisions can be perceived as a sensitive topic. As a result, participants might have downplayed the influence of financial incentives on their decision making, as it might conflict with professional ethics. Also, it might be challenging to analyse the impact of incentives on their own behaviour for participants who have used this system for many years and therefore may take the system for granted.

The findings on GPs’ awareness of profitability are in line with, and provide new knowledge to, existing research, such as a study from Sweden that found that GPs are aware of and adapt to incentives [20] However, in Sweden, GPs do not have financial incentives directly affecting their personal income; instead they operate at the clinic level. Our study contributes with knowledge of GPs’ perceptions in a system where GPs are self-employed, and their daily decisions directly affect their personal income. There was significant awareness of tasks that are perceived as poorly compensated or not compensated in the fee system. Although they receive capitation for each patient on the list, many expressed frustrations for not receiving remuneration from the fee system for certain tasks. Additionally, the participants believed that they themselves and a large majority of the GPs underused the fees. Both of these experiences may create a feeling of receiving less income than the workload would suggest. Therefore, the fee-for-service system may create a gap between GPs’ perceived potential income based on workload and their actual income.

The findings in this paper are relevant for the question whether fee-for-service system increase supplier induced demand [31]. For example, a study from Netherlands finds that fee-for-service increase the supplier-induced demand [32], while a study from Ireland does not finds that effect [33]. This paper cannot estimate an effect of fee-for-service but can contribute with a relevant theoretical basis. The results from this study shows that participants are aware of the profitability and acknowledge the influence on their behaviour. This supports the theoretical basis that fee-for-service have a potential to increase supplier-induced demand.

### Implications

The findings in our study may have several implications. First, the relationship between short consultation duration and GPs’ gatekeeping decisions should be taken into consideration when choosing payment model. Fee-for-service is often considered the most efficient model since it enables primary care to increase service provision. However, if fee-for-service leads to increased unnecessary treatments, it is not necessarily the most efficient payment model at a societal level. Such increased service provision should be balanced against other payment models’ potential lower provision of unnecessary treatments and interventions. Future research should be directed towards examining the presence and magnitude of this relationship.

Second, since GPs are aware of and may adapt to financial incentives, fee-for-service can be used as a management tool that are likely to influence what GPs do. As detailed in the result section, one of the participants described the influence of incentives in the following way: “what we do can generally be influenced by increasing or decreasing the fee”. Last, a large and complex system of fees has to be remembered and interpreted by the GPs and may cause underuse and overuse of fees. Policymakers should balance the need for targeted fees to incentivise certain desired procedures or behaviour against a simplification of the fee system that can lead to more correct use of public funding.

### Strengths and limitations

This focus group study has both strengths and limitations. We interviewed 33 GPs from 6 offices across Norway with differences in experience, age, and sex. Due to pragmatic reasons, participants were remunerated NOK 2000, interviews were conducted in GP offices with at least four GPs, and the focus group participants did work together at this office. To limit the influence of remuneration for recruitment and participant behaviour, we aimed to set the remuneration at a cost-neutral level for the participants. Also, the discussions would likely have unfolded differently if the participants had not known each other in front. However, by discussing the topics with colleagues in a familiar environment may have made the participants more willing to share their honest opinion. On the other hand, existing group dynamics, social hierarchies and norms at the workplace can also have constrained their possibility to speak freely. However, we were able to detect a patterned response on several themes that was largely consistent across the focus groups. In other words, the discussions developed in a similar way in most of the focus groups. Furthermore, this method’s strengths lie in exploring how participants’ discussions and discourse on the topic, rather than uncovering each individual’s in-depths attitudes. In-depth interviews with GPs without influence of group dynamics could be a fruitful way forward to enhance the understanding. Additionally, this method only captures what the participants say they do, and not necessarily what they actually do. Therefore, these questions should also be examined by using administrative register data.

The researchers involved in this paper have diverse educational backgrounds including social science, medicine, and other health educations. Therefore, the researchers’ positions and preconceptions are varied. The researchers who conducted the interviews, KBK and EHH, are both social scientists and do not themselves have experience with GP practice. This can be both a limitation and an advantage. Their preconceptions and previous knowledge may limit the ability to understand all aspects of GPs’ various tasks during the day, which could result in more misunderstandings or wrong interpretations than if the interviewers were GPs themselves. However, it may also be an advantage that the interviewers are not GPs; they do not interpret the participants in the light of their own GP experience and have an outsider perspective on the discussions. KØ and MN are medical doctors (GP and professor in community medicine, respectively), which may limit the potential for false interpretations. Also, the analysis of the material was mainly conducted by the first author (KBK), which can be a limitation to the study. However, to limit the shortcomings that may arise from this, the iterative process of reviewing themes involved thorough discussions with the co-authors. This study is part of a larger research project (#303583) that aims to examine potential side-effects of fee-for-service using national register data. Some of the researchers have publicly expressed opinions about the system. This project’s perspective will influence the researchers’ preconceptions of the topic and the design of the interview guide. In the data analysis, the researchers have endeavoured to highlight various perspectives to avoid cherry picking of results.

## Conclusions

GPs are aware of the profitability in the fee-for-service system, believe that fee-for-service may influence some of their decisions, and have challenges with remembering and interpreting certain fees. Furthermore, GPs’ gatekeeping decisions can be challenged by the fee-for-service system through incentivising shorter consultations and thereby providing less time needed to reject unnecessary treatments or interventions.

### Supplementary Information

**Supplementary Material 1. **

**Supplementary Material 2. **

## Data Availability

The dataset analysed during the current study is not publicly available due to participant privacy but may be available from the corresponding author on reasonable request.

## Abbreviations

GP: General practitioner
OECD: The Organisation for Economic Co-operation and Development
