# Supplementary material for: Time is money: general practitioners’ reflections on the fee-for-service system
Source: BMC Health Serv Res. 2024 Apr 15;24:472. doi: 10.1186/s12913-024-10968-3 (PMC11020312; doi:10.1186/s12913-024-10968-3)
Supplement: Supplementary file 1 — Supplementary Material 1. [file 12913_2024_10968_MOESM1_ESM.docx]

## Supplementary file 1– interview guide

Q1: What are the main advantages and disadvantages of being a self-employed general practitioner compared to a general practitioner who is employed by the municipality with a fixed salary?

Q2: Can you tell us a bit about how you organize your day at the medical office? For example, how much time do you allocate for each consultation, do you have a time buffer, and what does your day look like?

- What are the reasons behind you planning decisions?
- Do you manage to stick to your schedule? And what does lead to delays?

Q3: Are there times when you are unsure about which fees to use?

- Can you give examples?
- What factors contribute to whether you choose one fee over another?

Q4: Some fees allow for discretion, which may lead GPs to use them differently. Do you believe there is a large difference between GPs in how to use fees in the same situation?

- Can you give examples?
- Why do you think GPs use the fees differently?

Q5: Are you inadequately compensated for certain types of procedures?

- Can you provide examples?

Q6: On the contrary, are there procedures that are well compensated?

- Can you provide examples?

Q6B: Does the municipality have opinions/impose restrictions on how you use the fees? [asked only to fixed salary GPs as alternative questions to Q5 and Q6]

- Do you think fixed salary GPs use less fees than self-employed GPs?

Q7: Do you think that GPs do some procedures less/more because it is poorly/well compensated?

- Do you do this yourself?

Q8: Sometimes, patients may request referrals, antibiotics, addictive drugs or sick leave without a medical indication. In such cases, what takes more time: granting the request or rejecting it?

- How long time does it take to say yes/no?

Q9: Do you feel that you are well or poorly compensated for the challenging conversations where you have to say no?
