# Supplementary material for: Time is money: general practitioners’ reflections on the fee-for-service system
Source: BMC Health Serv Res. 2024 Apr 15;24:472. doi: 10.1186/s12913-024-10968-3 (PMC11020312; doi:10.1186/s12913-024-10968-3)
Supplement: Supplementary file 2 — Supplementary Material 2. [file 12913_2024_10968_MOESM2_ESM.docx]

## Supplementary file 2 – selected fees

| **Fee** | **Norwegian original text** | **English translation (by the authors)** |
| --- | --- | --- |
| 615 | Samtaleterapi ved allmennlege med minst 15 minutters varighet med pasienter med psykiske lidelser. Samtalen må avvike fra en vanlig samtale om medisinske problemstillinger, og være av terapeutisk karakter. | Talking therapy with GP with at least 15 minutes duration with patients with psychological disorders. The talking therapy must differ from an ordinary talk about medical questions and be of therapeutic character. |
| 2ld | Systematisk legemiddelgjennomgang hos fastlege. Systematisk legemiddelgjennomgang i tråd med Helsedirektoratets veileder, herunder bruk av relevant beslutningsstøtte og innhenting av informasjon fra andre enn pasienten i nødvendig grad (hjemmesykepleie, pårørende, spesialisthelsetjeneste). Kan kun kreves for egne listeinnbyggere med behov for langvarig behandling med fire legemidler eller mer når fastlege finner dette nødvendig ut fra en medisinsk vurdering. Vurdering av behov, hvem som er konsultert og iverksatte tiltak skal fremgå av pasientjournalen. Taksten kan benyttes inntil tre ganger pr kalenderår pr. pasient. | Systematic Medication Review by general practitioner. Systematic medication review in accordance with the guidelines of the Directorate of Health, including the use of relevant decision-making support and gathering information from sources other than the patient to the extent necessary (home care, next of kin, specialist services). This can only be used for patients listed to the general practitioner with a need for long-term treatment with four or more medications when the general practitioner consider it necessary based on a medical assessment. The assessment of the need, who has been consulted, and implemented measures needs to be documented in the patient's journal. This fee can be used up to three times per calendar year per patient. |
| 102 | Oppstart (første konsultasjon) av individuell strukturert røykeavvenning som ledd i behandling av sykdom, i henhold til Nasjonal faglig retningslinje for røykeavvenning (https://helsedirektoratet no/retningslinjer/roykeavvenning) Taksten kan benyttes inntil to ganger per pasient per kalenderår. Kan ikke benyttes på legevakt | Initiation (first consultation) of individual structured smoking cessation as part of the treatment for a disease, in line with the National clinical guideline for smoking cessation (https://helsedirektoratet.no/retningslinjer/roykeavvenning). The fee can be used up to two times per patient per calendar year. It cannot be used for out-of-hours clinic. |
| 111 | Kryokirurgi. Takst 10c kan kreves én gang selv om taksten repeteres | Cryosurgery. Fee 10c can be used once even if this fee is repeated. |
| 2AEK | E-konsultasjon hos fastlege (kveld) | Electronic consultation with a general practitioner (evening) |
| 129f | Ambulant kontinuerlig 24 timers registrering av blodtrykk Tyding, teknisk assistanse, bruk av utstyr m v | Ambulatory continuous 24-hour blood pressure monitoring. Interpretation, technical assistance, use of equipment, etc. |
| 2CD | Tillegg for tidsbruk ved konsultasjonsvarighet utover 20 min per påbegynt 15 min | Additional fee for consultation duration exceeding 20 minutes per started 15 minutes." |
